# Hemorheological, cardiorespiratory, and cerebrovascular effects of pentoxifylline following acclimatization to 3,800 m

**DOI:** 10.1152/ajpheart.00783.2023

**Published:** 2024-01-19

**Authors:** Andrew R. Steele, Connor A. Howe, Travis D. Gibbons, Katharine Foster, Alexandra M. Williams, Hannah G. Caldwell, L. Madden Brewster, Jennifer Duffy, Justin A. Monteleone, Prajan Subedi, James D. Anholm, Mike Stembridge, Philip N. Ainslie, Joshua C. Tremblay

**Affiliations:** ^1^Centre for Heart, Lung & Vascular Health, School of Health and Exercise Sciences, University of British Columbia-Okanagan, Kelowna, British Columbia, Canada; ^2^Department of Biological Sciences, Northern Arizona University, Flagstaff, Arizona, United States; ^3^Pulmonary and Critical Care, Veterans Affairs Loma Linda Healthcare System, Loma Linda, California, United States; ^4^Department of Medicine, Loma Linda University School of Medicine, Loma Linda, California, United States; ^5^Department of Cellular & Physiological Sciences, Faculty of Medicine, University of British Columbia, Kelowna, British Columbia, Canada; ^6^Cardiff School of Sport and Health Sciences, Cardiff Metropolitan University, Cardiff, United Kingdom

**Keywords:** cerebral blood flow, chemoreflex, high altitude, hypoxic pulmonary vasoconstriction, red blood cell

## Abstract

Pentoxifylline is a nonselective phosphodiesterase inhibitor used for the treatment of peripheral artery disease. Pentoxifylline acts through cyclic adenosine monophosphate, thereby enhancing red blood cell deformability, causing vasodilation and decreasing inflammation, and potentially stimulating ventilation. We conducted a double-blind, placebo-controlled, crossover, counter-balanced study to test the hypothesis that pentoxifylline could lower blood viscosity, enhance cerebral blood flow, and decrease pulmonary artery pressure in lowlanders following 11–14 days at 3,800 m. Participants (6 males/10 females; age, 27 ± 4 yr old) received either a placebo or 400 mg of pentoxifylline orally the night before and again 2 h before testing. We assessed arterial blood gases, venous hemorheology (blood viscosity, red blood cell deformability, and aggregation), and inflammation (TNF-α) in room air (end-tidal oxygen partial pressure, ∼52 mmHg). Global cerebral blood flow (gCBF), ventilation, and pulmonary artery systolic pressure (PASP) were measured in room air and again after 8–10 min of isocapnic hypoxia (end-tidal oxygen partial pressure, 40 mmHg). Pentoxifylline did not alter arterial blood gases, TNF-α, or hemorheology compared with placebo. Pentoxifylline did not affect gCBF or ventilation during room air or isocapnic hypoxia compared with placebo. However, in females, PASP was reduced with pentoxifylline during room air (placebo, 19 ± 3; pentoxifylline, 16 ± 3 mmHg; *P* = 0.021) and isocapnic hypoxia (placebo, 22 ± 5; pentoxifylline, 20 ± 4 mmHg; *P* = 0.029), but not in males. Acute pentoxifylline administration in lowlanders at 3,800 m had no impact on arterial blood gases, hemorheology, inflammation, gCBF, or ventilation. Unexpectedly, however, pentoxifylline reduced PASP in female participants, indicating a potential effect of sex on the pulmonary vascular responses to pentoxifylline.

**NEW & NOTEWORTHY** We conducted a double-blind, placebo-controlled study on the rheological, cardiorespiratory and cerebrovascular effects of acute pentoxifylline in healthy lowlanders after 11–14 days at 3,800 m. Although red blood cell deformability was reduced and blood viscosity increased compared with low altitude, acute pentoxifylline administration had no impact on arterial blood gases, hemorheology, inflammation, cerebral blood flow, or ventilation. Pentoxifylline decreased pulmonary artery systolic pressure in female, but not male, participants.

## INTRODUCTION

Exposure to high altitude prompts a cascade of integrated physiological responses that aim to mitigate the decline in oxygen partial pressure and maintain oxygen delivery (1–3). However, rapid ascent to high altitude can result in high altitude-related health problems, including acute mountain sickness and two life-threatening diseases: high-altitude pulmonary edema and high-altitude cerebral edema (4, 5). These high-altitude illnesses are often associated with heightened whole blood viscosity (6), elevated pulmonary vascular pressure (5, 7), and increased systemic oxidative-inflammatory stress (8, 9).

Hypobaric hypoxia stimulates plasma volume decreases and erythropoiesis, leading to a rapid and sustained increase in whole blood viscosity (10), which can impede microvascular flow (11, 12). Impaired red blood cell deformability independent of hemoconcentration may also contribute to this detriment in microcirculation flow (13). Acute hypoxia causes a decrease in red blood cell deformability in a dose-dependent manner (14), occurring with greater red blood cell oxidative stress (15). Furthermore, lowlanders during ascent to high altitude (1,350 to 5,050 m) had a significant and progressive reduction in red blood cell deformability, which correlated with an increase in altitude (13). We therefore reasoned that hypoxic exposure would decrease red blood cell deformability.

Pentoxifylline, a rheologically active drug used for the treatment of intermittent claudication in peripheral artery disease (16, 17), may offer potential therapeutic benefits at high altitude. For example, pentoxifylline (400 mg, 3 times daily) prevented an increase in whole blood viscosity and attenuated a decline in red blood cell filtration (a surrogate of red blood cell deformability) during a 3-wk high-altitude expedition at 5,150 m (18). Acute pentoxifylline [bolus, 100 mg intravenously (iv)] lowers pulmonary artery pressure in individuals with chronic cor pulmonale during both rest and exercise and it also attenuates the hypoxic pulmonary vasoconstrictive response in dogs (19). Pentoxifylline’s potential to lower pulmonary artery pressure and reduce hypoxic vasoconstrictive responsiveness at high altitude might be attributed to its capacity to lower blood viscosity (20), induce pulmonary vasodilation (21), and decrease inflammation (22).

Pentoxifylline has also been observed to increase global and regional cerebral blood flow in both healthy participants and patients with cerebrovascular disease (23). Although the exact mechanism is still debated, current evidence suggests that pentoxifylline primarily enhances cerebral blood flow by inducing hemorheological changes (improved red cell deformability), thereby promoting improved cerebral microcirculation flow (24–26). In addition, pentoxifylline (single dose, 400 mg po) has demonstrated its ability to prevent decreases in cerebral blood flow during rapid exposure to stimulated 4,000 m using a decompression chamber (27). However, pentoxifylline (400 mg, once daily for 5 wk) has also been associated with an increase in arterial oxygen saturation ($\text{S}{\text{a}}_{{\text{O}}_{2}}$) at high altitude following 5 wk at 4,600 m (28), which would oppose further dilation since arterial oxygen content is tightly linked with cerebral blood flow [as reviewed in Willie et al. (29)]. The precise mechanism underlying this augmented arterial oxygen saturation remains unknown, although it is plausible that pentoxifylline could stimulate ventilation like other methylxanthines (e.g., caffeine, theophylline, and aminophylline) (30), and even mild hyperventilation is an effective means to increase oxygen saturation in chronic hypoxia.

Despite these promising findings, the impact of pentoxifylline on integrated physiology responses and its potential therapeutic use at high altitude have not been explored. Herein, we conducted a comprehensive assessment of blood rheology, pulmonary artery pressure, ventilation, inflammation, and cerebral blood flow using a double-blind placebo-controlled research study in young healthy male and female volunteers sojourning to high altitude (3,800 m) following 11–14 days of acclimatization. We hypothesized that pentoxifylline would lower whole blood viscosity, reduce pulmonary artery pressure, and decrease cerebral blood flow by increasing ventilation.

## METHODS

### Ethical Approval

All experimental procedures had Institutional Review Board approval from the University of British Columbia (H22-01091) and conformed to the latest revision of the Declaration of Helsinki, except for registration in a database. Before participation, all subjects provided written informed consent. This study was part of a larger research expedition conducted in September 2022 at the Barcroft Field Station (White Mountain, CA; 3,800 m). As such, participants took part in a number of studies conducted at the Barcroft Field Station. However, the a priori, primary research questions addressed in the current paper are novel and are exclusively dealt with in this study alone; there was no overlap between this investigation and others completed on the research expedition.

### Participants

Sixteen participants were recruited, including 10 females and 6 males, with an average age of 27 ± 5 yr (means ± SD). They were normotensive (systolic blood pressure < 140 and diastolic pressure < 90 mmHg) and had a body mass index ≤ 30 kg·m^−2^. Participants were nonsmokers, who had no previous history of cardiovascular, cerebrovascular, or respiratory diseases. All testing occurred at high altitude. However, we additionally made hemorheological measures at low altitude (see *Measurements*). Low-altitude testing occurred at the University of British Columbia-Okanagan Campus in British Columbia (altitude, ∼344 m) 1 mo before departure to high altitude. All participants arrived at Barcroft Field Station on the same day and approximately at the same time (i.e., within 1 h). Participants rapidly ascended to 3,800 m by motor vehicle over the course of 2 or 3 h. Testing took place between the 11th and 14th day after arrival at 3,800 m. Participants abstained from prophylactic altitude sickness drugs such as acetazolamide and corticosteroids. Participants refrained from using nonsteroidal anti-inflammatories for at least 24 h before the experiments. Before each experiment, all participants abstained from alcohol and caffeine for ≥24 h. Participants were sea-level residents and had not traveled to high altitude within 6 mo before the study. None of the participants was experiencing any symptoms of acute mountain sickness, as determined by the Lake Louise Questionnaire.

### Experimental Design

**Figure 1.**
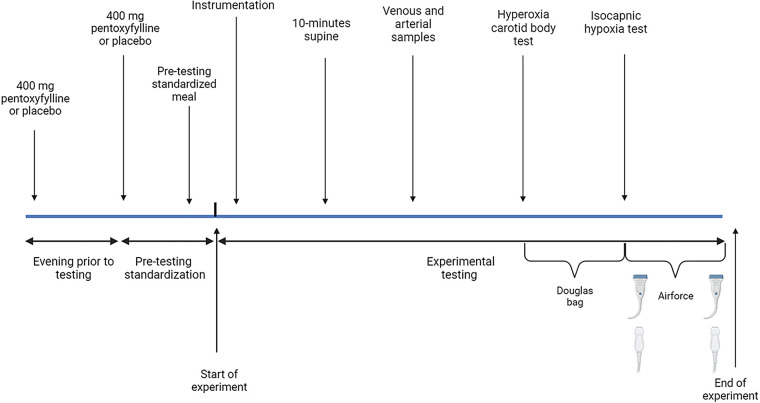
Schematic of the experimental design. Participants were given 400 mg of pentoxifylline or placebo the night prior and again 2 h before testing began. The experiment began with participants laying in supine and resting state quietly for ∼10 min before collections of resting venous blood samples and radial artery blood samples. Following this, we assessed carotid body chemoreflex-mediated inhibition of ventilation (hyperoxia carotid body test) using a Douglas bag. Thereafter, we assessed carotid body chemoreflex-mediated activation of ventilation (isocapnic hypoxia test) participants using an end-tidal forcing system to maintain end-tidal carbon dioxide partial pressure ($\text{P}E{\text{T}}_{\text{C}{\text{O}}_{2}}$) at baseline values, while being exposed to 2 progressive hypoxic stages [end-tidal partial pressure of oxygen ($\text{P}E{\text{T}}_{{\text{O}}_{2}}$) = 45 and 40 mmHg]. Echocardiography and cerebral blood flow were measured at both baseline and during the final minute of the last stage of isocapnic hypoxia.

A schematic of the experimental design is presented in Fig. 1. We used a counterbalanced, crossover, double-blind, placebo-controlled design. Participants were tested on 2 different days with 40 h between testing conditions. Participants received pentoxifylline or a placebo on any given day, without a predetermined sequence. However, we used a counterbalanced design to mitigate the impact of day or sequence effects on physiological outcomes. Randomization and drug coordination were conducted by an external member of our research team who was not involved in data collection. This approach guaranteed complete blinding of both participants and researchers to the specific condition being administered. Participants received either a placebo or 400 mg of sustained-release generic pentoxifylline (Apotex, Toronto, Canada) tablets orally on the night before testing and again 2 h before the test (total, 800 mg). Sustained-release tablets are rapidly absorbed, and blood concentrations peak within 3 h and remain elevated for 14 h (31). Testing commenced between 0600 and 1400 h and was matched for time of day between conditions. Participants received a standardized meal 30 min before testing consisting of one Clif bar (Mondelez International). The experiment began with participants lying in supine and resting positions quietly for ∼10 min before collections of resting venous blood samples and radial artery blood samples (see *Measurements*).

Following this, we assessed carotid body tonic activity as chemoreflex-mediated inhibition of ventilation. As previously described (32, 33), 100% oxygen via a Douglas bad was delivered for 1 min followed by ∼3 min of room air breathing or until end-tidal oxygen partial pressure ($\text{P}E{\text{T}}_{{\text{O}}_{2}}$) returned to baseline. This process was repeated four times. The two or three breath nadir in response to hyperoxia was noted, and the average of the four trials was used to provide an index of the carotid body’s contribution to resting room-air ventilation. Subsequently, we assessed carotid body-mediated activation of ventilation by progressive isocapnic hypoxia. Participants were connected to an end-tidal forcing system (see *Measurements*) to maintain carbon dioxide partial pressure ($\text{P}E{\text{T}}_{\text{C}{\text{O}}_{2}}$) at baseline values, while being exposed to two progressive hypoxic stages. Isocapnic hypoxia stages were clamped at $\text{P}E{\text{T}}_{{\text{O}}_{2}}$ = 45 and 40 mmHg. Before isocapnic hypoxia, participants were initially clamped at their room-air $\text{P}E{\text{T}}_{\text{C}{\text{O}}_{2}}$ and $\text{P}E{\text{T}}_{{\text{O}}_{2}}$ for a 10-min baseline period to collect baseline echocardiography and cerebral blood flow. Each hypoxic stage consisted of 3 min of steady-state breathing, with echocardiography and cerebral blood flow scans recollected after this 3-min period during the last hypoxic stage (40 mmHg). The last minute of each hypoxic stage ventilation was collected and a linear slope between ventilatory variables and peripheral oxygen saturation was used to determine the hypoxic ventilatory response. Pulmonary arterial systolic pressure reactivity was determined by taking the last minute of 40-mmHg isocapnia for $\text{P}E{\text{T}}_{{\text{O}}_{2}}$ and pulmonary arterial systolic pressure relative to room air:

$$\;\text{Pulmonary arterialsystolic pressure (PASP) reactivity = }\left(\frac{\text{ΔPASP}}{\text{Δ}\text{P}E{\text{T}}_{{\text{O}}_{2}}}\right)\text{ = }\left(\frac{{\left(\text{PASP}\right)}_{\text{room air}}-{\left(\text{PASP}\right)}_{\text{isocapnic hypoxia (40 mmHg)}}}{{\left(\text{P}E{\text{T}}_{{\text{O}}_{2}}\right)}_{\text{room air}}-{\left(\text{P}E{\text{T}}_{{\text{O}}_{2}}\right)}_{\text{isocapnic hypoxia (40 mmHg)}}}\right)$$

### Measurements

#### Transthoracic echocardiography.

Echocardiography was conducted using ultrasound (Vivid iq, GE Healthcare, Piscataway, NJ) and a phased array transducer (1.5- to 3.6-MHz M4S-RS, GE Healthcare, Piscataway, NJ) by a trained sonographer (A.M.W.). A three-lead electrocardiograph was connected to the participant to enable cardiac cycle gating during the examination. With the participant in the left lateral decubitus position, images were acquired for the assessment of cardiac function according to current guidelines (34). The imaging was obtained at end expiration for five consecutive cardiac cycles, and the data were saved for later offline analysis using EchoPAC software (GE Medical, Horton, Norway). Measurements were taken in triplicate from consecutive cardiac cycles and then averaged to enhance accuracy. To assess the diameter of the inferior vena cava, subcostal images were acquired while the participant was lying in supine position. Cardiac output was calculated as stroke volume (mL) × heart rate (beats/min), and total peripheral resistance was calculated as mean arterial pressure (mmHg)/cardiac output (mL/min). PASP was quantified as the maximum systolic pressure gradient across the tricuspid valve added to right atrial pressure estimated from the collapsibility of the inferior vena cava. To derive pressure, the modified Bernoulli equation (4*V*^2^) was applied to the peak systolic regurgitation jet velocity measured via continuous wave Doppler.

#### Cerebral blood flow.

Diameter and blood velocity of the right vertebral artery and the right internal carotid artery were measured using a 10-MHz multifrequency linear array duplex ultrasound (Terason uSmart 3300, Teratech, Burlington, MA). Scans of the vertebral artery and internal carotid artery were performed sequentially. Diameter and peak blood velocity were simultaneously measured using B-mode and pulse-wave mode, respectively. The insonation angle was 60°. For the internal carotid artery, diameter measurements were taken at a location at least 1.5 cm distal to the common carotid bifurcation to avoid capturing turbulent flow. Vertebral artery flow was always measured from the same intervertebral space within participants. Diameter and peak blood flow velocity were captured using screen capture software and subsequently stored for offline analysis. Peak blood velocity and diameter were analyzed at 30 Hz with a custom wall edge tracking software (35). One-minute averages were collected for internal carotid artery and vertebral artery blood flow at baseline and at the last minute of isocapnic hypoxia. Volumetric blood flow was calculated as

$$\text{Volumetric blood flow }=\;\left(0.5\;\times \text{peak envelope velocity}\right)\times [\text{π}{\left(0.5\;\times \text{diameter}\right)}^{2}]\;\times 60$$

Global cerebral blood flow was calculated as twice the sum of the internal carotid artery and the vertebral artery.

#### Cardiorespiratory metrics.

Systolic and diastolic blood pressures were measured using an automated cuff sphygmomanometer (CARESCAPE V100, GE Healthcare). Mean arterial pressure was subsequently calculated as follows: (1/3 systolic blood pressure) + (2/3 × diastolic blood pressure). Arterial oxygen saturation ($\text{S}{\text{p}}_{{\text{O}}_{2}}$) was estimated by pulse oximetry (Rad-5, Masimo) using the index finger sensor. $\text{P}E{\text{T}}_{{\text{O}}_{2}}$ and $\text{P}E{\text{T}}_{\text{C}{\text{O}}_{2}}$ were collected continuously using a gas analyzer (model ML206, ADInstruments). $\text{P}E{\text{T}}_{\text{C}{\text{O}}_{2}}$ and $\text{P}E{\text{T}}_{{\text{O}}_{2}}$ were detected using a peak detection analysis. Respiratory flow was continuously measured by a pneumotachometer (model MLT1000L, ADInstruments). The gas analyzer and pneumotachometer were calibrated before daily testing and between participants. $\text{P}E{\text{T}}_{\text{C}{\text{O}}_{2}}$, $\text{P}E{\text{T}}_{{\text{O}}_{2}}$, and minute ventilation were recorded at 1,000 Hz and integrated with a data acquisition system (PowerLab 16/30, ADInstruments) and stored for subsequent analysis using associated software (LabChart 8.0 Pro, ADInstruments). A reactivity metric was calculated for hemodynamic variables, respiratory variables, and cerebral blood flow by determining the delta change between room-air conditions and isocapnic hypoxia for each day.

#### Dynamic end-tidal forcing.

Both $\text{P}E{\text{T}}_{\text{C}{\text{O}}_{2}}$ and $\text{P}E{\text{T}}_{{\text{O}}_{2}}$ were controlled using a dynamic end-tidal forcing system that has been previously described in detail (36). End-tidal forcing targets breath-by-breath end-tidal $\text{P}E{\text{T}}_{\text{C}{\text{O}}_{2}}$ and $\text{P}E{\text{T}}_{{\text{O}}_{2}}$, thereby controlling arterial gases independent of ventilation. Our end-tidal forcing system controls end-tidal gases through a wide range of $\text{P}E{\text{T}}_{\text{C}{\text{O}}_{2}}$ and $\text{P}E{\text{T}}_{{\text{O}}_{2}}$ and has been used for similar $\text{P}E{\text{T}}_{{\text{O}}_{2}}$ manipulations (36).

### Blood Collection

Radial artery blood samples (2 mL) were collected using a lithium heparin-coated autofill syringe. Immediately following sample collection, any air bubbles were evacuated from the syringe before analysis. Blood analysis, including pH, arterial partial pressure of carbon dioxide ($\text{P}{\text{a}}_{{\text{CO}}_{2}}$), arterial partial pressure of oxygen ($\text{P}{\text{a}}_{{\text{O}}_{2}}$), arterial oxygen saturation ($\text{S}{\text{a}}_{{\text{O}}_{2}}$), lactate, glucose, sodium, and potassium, was performed in duplicate within 2 min of sampling using a commercially available gas analyzer (ABL90FLEX PLUS, Radiometer). The gas analyzer was calibrated every 8 h using the manufacturer’s standard internal quality checks. Hematocrit was obtained by the micromethod (PowerSpin BX Centrifuge-unico; 10 min at 13,000 rpm). Arterial oxygen content ($\text{C}{\text{a}}_{{\text{O}}_{2}}$) was calculated from [Hb] × 1.34 × ($\text{S}{\text{p}}_{{\text{O}}_{2}}$/100) + (0.003 × $\text{P}{\text{a}}_{{\text{O}}_{2}}$).

Venous blood samples were obtained from the antecubital vein using K2 EDTA tubes (12 mL). Following collection, venous samples were promptly centrifuged, aliquoted, and flash-frozen (−196°C). Samples were stored at −80°C until analysis at our laboratory in Kelowna, British Columbia.

### Hemorheological and Biochemical Analysis

Whole blood viscosity was measured in duplicate within 15 min of blood sample acquisition at seven shear rates of 47.5, 67.5, 90, 150, 225, 337.5, and 450·s^−1^ at 37°C with a cone-and-plate viscometer (Model DV2T, CPA-40Z spindle; Brookfield AMETEK, Middleboro, MA). These shear rates were selected based on the shear-thinning properties of erythrocytes and the heterogeneity of shear rates within human conduit arteries (37). To isolate plasma viscosity from whole blood viscosity, whole blood was centrifuged at 600 *g* and 4°C for 10 min. The viscosity of the separated plasma was then measured at two shear rates 225 and 450·s^−1^ at 37°C. The selection of these shear rates was based on their physiological relevance, representing conditions that closely mimic physiological stresses. Red blood cell deformability was analyzed using laser-assisted optical rotational red cell analyzer (LORRCA, RR Mechatronics, Hoorn, The Netherlands) (38). Whole blood (∼14 µL) was dissolved in 5 mL of isotonic 0.14 mM polyvinylpyrrolidone (osmolarity 300 mosmol/L, viscosity 28.38 mPa·s at 37°C). About 880 uL of sample was then placed into the Couette system (bob) and sheared at nine different rates between 0.3 and 30 Pa. A laser beam is directed through the samples during shearing and deformation of red blood cells was determined based on the diffraction pattern of the laser beam. The LORRCA software used width (*W*) and length (*L*) of the diffraction pattern to calculate an elongation index (EI): EI = (*L* − *W*)/(*L* + *W*) for the nine shear stresses. The Lineweaver-Burk model (commonly used for the enzyme activity equation) was used to determine the maximum red cell deformation (EI_max_), representing a theoretical maximal elongation index and the shear stress at half maximal deformation (SS½) as previously described (39). Furthermore, we calculated the ratio between SS½ and EI_max_ as an additional metric for determining alternations of red blood cell deformability. The ratio between SS½ and EI_max_ is a more robust metric for determining alternations in red blood cell deformability compared with SS½ and EI_max_ alone (40). A greater SS½:EI_max_ reflects worse deformability.

The red blood cell aggregation was also performed by LORRCA. The computer program analyzed the aggregation parameters of red blood cells, which were based on the syllectogram, the curve of relation between intensity of laser backscattered and time. Red blood cell aggregation was quantified as aggregation index (AI) as previously described (38).

Plasma concentration of tumor necrosis factor-α (TNF-α) was quantified using a sandwich solid-phase enzyme-linked immunosorbent assay (R&D, Cat. No. HSTA00E).

### Statistical Analysis

Statistical analyses were performed using the statistical language R (R-4.3.1 Foundation for Statistical Computing). Figures were created using GraphPad, Prism 8.3.0 (GraphPad Software). All data are presented as means ± SD with statistical significance set at *P* < 0.05. Data were assessed for normality and variance using the Shapiro–Wilk and the Bron–Forsythe tests. We used paired two-tailed Student’s *t* tests to compare placebo and pentoxifylline (and for hemorheology, low altitude vs. placebo) for various parameters, including demographics, blood gases, electrolytes, whole blood, plasma viscosity, TNF-α levels, carotid body chemoreflex-mediated inhibition of ventilation, and carotid body chemoreflex-mediated activation of ventilation. In addition, we used a paired two-tailed Student’s *t* tests for these comparisons: placebo room air versus pentoxifylline room air, placebo isocapnic hypoxia versus pentoxifylline isocapnic hypoxia, and between-day reactivity for hemodynamic variables, respiratory variables, cerebral blood flow, and pulmonary artery systolic pressure. The measured differences for global cerebral blood flow and internal carotid artery blood flow during isocapnic hypoxia were not normally distributed; therefore, comparisons were made using a Wilcoxon matched-pairs signed-rank test.

We also used an exploratory linear mixed-effects model with pulmonary artery systolic pressure as the dependent variable. In this analysis, we included drug (placebo vs. pentoxifylline) and sex as fixed factors, with participant identifiers serving as the random factor. Our aim was to identify any potential interactions among drug and sex that may have existed. When significant interactions were observed, we conducted within-sex comparisons for pulmonary artery systolic pressure using paired two-tailed Student’s *t* tests. To gauge effect size, we calculated Cohen’s *d* using the following formula:

Cohen′s d= (mean of group 1−mean of group 2)/pooled SD

$$\text{Pooled SD }=\;\surd \{\left[\left({\text{SD 1}}^{2}\right)\;+\;\left({\text{SD 2}}^{2}\right)\right]/\text{2}\}$$

## RESULTS

### Blood Gases and Rheology

Standard blood gas metrics (pH, $\text{P}{\text{a}}_{{\text{CO}}_{2}}$, $\text{P}{\text{a}}_{{\text{O}}_{2}}$, ${\text{HCO}}_{3}^{-}$, and $\text{S}{\text{a}}_{{\text{O}}_{2}}$) and inflammation (TNF-α) were not different between days (see Table 1). TNF-α coefficient of variation was 4.7%. Whole blood viscosity, plasma viscosity, and red blood cell deformability at all shear rates were not different with pentoxifylline compared with placebo. EI_max_, SS½, SS½:EI_max_, and aggregation index were also all not different between days (see Table 2). Compared with low altitude, whole blood viscosity and hematocrit, but not plasma viscosity, were elevated. In addition, SS½ and SS½:EI_max_ were elevated at high altitude, indicating worsened deformability, but there was no change in EI_max_ or aggregation index (see Table 3).

**Table 1.** Arterial blood gases, electrolytes, and venous blood inflammation

	Placebo	Pentoxifylline	*P* Value
pH	7.45 ± 0.02	7.46 ± 0.02	0.13
$\text{P}{\text{a}}_{{\text{CO}}_{2}}$, mmHg	29 ± 2	29 ± 2	0.83
$\text{P}{\text{a}}_{{\text{O}}_{2}}$, mmHg	59 ± 3	58 ± 4	0.65
${\text{HCO}}_{3}^{-}$, mmol/L	22 ± 1	23 ± 1	0.28
$\text{S}{\text{a}}_{{\text{O}}_{2}}$, %	91 ± 2	91 ± 2	0.70
Lactate, mmol/L	0.7 ± 0.2	0.7 ± 0.2	0.91
$\text{C}{\text{a}}_{{\text{O}}_{2}}$	19 ± 2	18 ± 2	0.57
Potassium, mmol/L	3.9 ± 0.6	3.8 ± 0.6	0.40
Sodium, mmol/L	138.9 ± 1.5	139.2 ± 1.5	0.26
Glucose, mmol/L	5.2 ± 0.8	5.0 ± 0.4	0.33
Osmolality, mmol/kg	283.2 ± 3.1	283.5 ± 3.1	0.58
TNF-α, pg/mL	1.2 ± 0.3	1.1 ± 0.3	0.64

Values are means ± SD unless otherwise specified; *n* = 14 participants. $\text{P}{\text{a}}_{{\text{CO}}_{2}}$, carbon dioxide arterial partial pressure; $\text{P}{\text{a}}_{{\text{O}}_{2}}$, oxygen arterial partial pressure; ${\text{HCO}}_{3}^{-}$, bicarbonate; $\text{S}{\text{a}}_{{\text{O}}_{2}}$ arterial saturation; $\text{C}{\text{a}}_{{\text{O}}_{2}}$, arterial content; TNF-α, tumor necrosis factor-α. A paired 2-tailed Student’s *t* tests were used for the statistical tests.

**Table 2.** Whole blood viscosity, plasma viscosity, red blood cell deformability, and aggregation

	Placebo	Pentoxifylline	*P* Value
Whole blood viscosity, mPa·s			
Shear rate of			
45·s^−1^	6.37 ± 0.65	6.54 ± 1.08	0.97
67.5·s^−1^	5.84 ± 0.62	5.68 ± 0.69	0.88
90·s^−1^	5.51 ± 0.57	5.35 ± 0.64	0.77
150·s^−1^	5.11 ± 0.49	4.96 ± 0.59	0.72
225·s^−1^	4.84 ± 0.46	4.70 ± 0.57	0.73
337.5·s^−1^	4.60 ± 0.50	4.47 ± 0.54	0.72
450·s^−1^	4.29 ± 0.34	4.31 ± 0.33	0.74
Plasma viscosity, mPa·s			
Shear rate of			
225·s^−1^	1.52 ± 0.11	1.47 ± 0.15	0.91
450·s^−1^	1.43 ± 0.08	1.44 ± 0.09	0.78
Hematocrit, %	48.2 ± 3.6	47.3 ± 3.5	0.13
Hemoglobin, g/dL	15.1 ± 1.5	14.8 ± 1.4	0.89
SS½	1.88 ± 0.26	1.9 ± 0.32	0.32
EI_max_	0.64 ± 0.012	0.64 ± 0.012	0.75
SS½:EI_max_	2.93 ± 0.46	2.96 ± 0.48	0.64
AI	66.7 ± 9.8	63.8 ± 10.7	0.21

Values are means ± SD unless otherwise specified; *n* = 14 participants. SS½, shear stress at half maximal deformation; EI_max_, maximal red blood cell deformation; AI, aggregation index. A paired 2-tailed Student’s *t* tests were used for the statistical tests.

**Table 3.** Hemorheology parameters measured at low altitude and during the placebo day of testing at high altitude

	Low Altitude	Placebo	*P* Value
Whole blood viscosity, mPa·s			
Shear rate of			
45·s^−1^	5.45 ± 0.59	6.37 ± 0.65	<0.01
67.5·s^−1^	5.01 ± 0.43	5.84 ± 0.62	<0.01
90·s^−1^	4.70 ± 0.36	5.51 ± 0.57	<0.01
150·s^−1^	4.30 ± 0.32	5.11 ± 0.49	<0.01
225·s^−1^	4.07 ± 0.33	4.84 ± 0.46	<0.01
337.5·s^−1^	3.90 ± 0.31	4.60 ± 0.50	<0.01
450·s^−1^	3.81 ± 0.32	4.29 ± 0.34	<0.01
Plasma viscosity, mPa·s			
Shear rate of			
225·s^−1^	1.45 ± 0.14	1.52 ± 0.11	0.085
450·s^−1^	1.39 ± 0.11	1.43 ± 0.08	0.087
Hematocrit, %	46.2 ± 3.0	48.2 ± 3.6	<0.01
SS½	1.70 ± 0.30	1.88 ± 0.26	0.01
EI_max_	0.63 ± 0.014	0.64 ± 0.012	0.61
SS½:EI_max_	2.68 ± 0.38	2.93 ± 0.46	0.03
AI	60.2 ± 14.2	66.7 ± 9.8	0.34

Values are means ± SD unless otherwise specified; *n* = 14 participants. SS½, shear stress at half maximal deformation; EI_max_, maximal red blood cell deformation; AI, aggregation index. A paired 2-tailed Student’s *t* tests were used for the statistical tests.

### Cardiorespiratory and Pulmonary Artery Systolic Pressure

**Figure 2.**
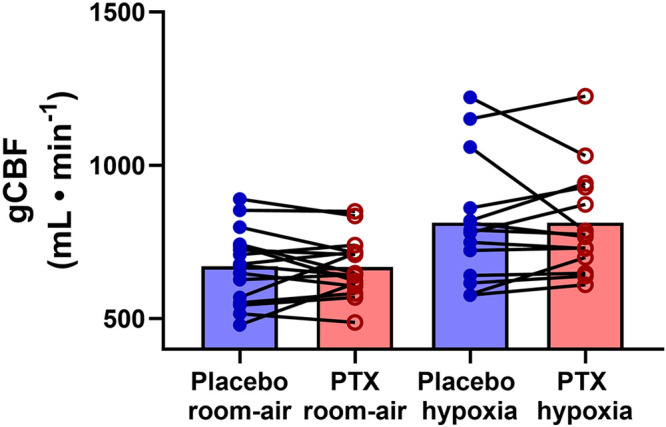
Global cerebral blood flow (gCBF) with placebo (blue) and pentoxifylline (PTX; red) during room air (*n* = 16) and isocapnic hypoxia (40 mmHg; *n* = 14). Closed dots are individual responses, and bar graphs are group means. Paired 2-tailed Student’s *t* tests were used for the room air comparisons, and a Wilcoxon matched-pairs signed-rank test was used for the hypoxia comparisons.

**Figure 3.**
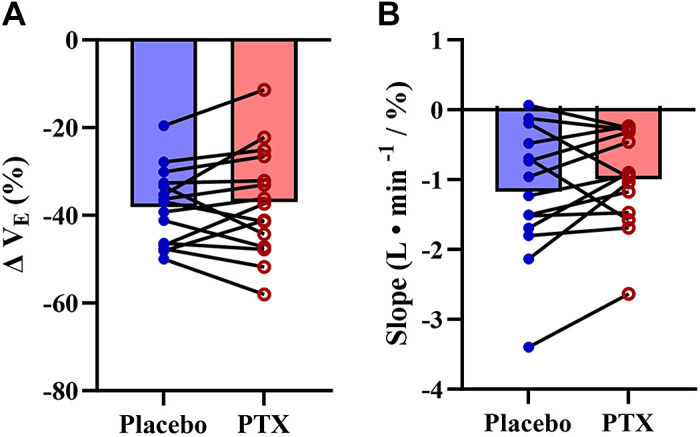
Carotid body tonic activity and sensitivity to hypoxia during placebo and pentoxifylline (PTX) conditions. *A*: carotid body tonic activity was assessed as the minute ventilation (V̇e) in response to transient hyperoxia reported as a relative reduction from baseline during placebo (blue) and PTX (red) (*n* = 15). *B*: carotid body sensitivity to hypoxia was assessed using isocapnic hypoxia during placebo and PTX (*n* = 14). To create a sensitivity slope, the minute ventilation of the last minute of each hypoxic stage (room air, end-tidal partial pressure of oxygen of 45 mmHg and end-tidal partial pressure of oxygen of 40 mmHg) was collected, and a linear slope between minute ventilation and arterial oxygen saturation was used to determine the acute hypoxic ventilatory response. Closed dots are individual responses, and bar graphs are group means.

There were no differences between experimental days for both room air and isocapnic hypoxia minute ventilation, cardiac output, mean arterial pressure (see Table 4), and global cerebral blood flow (room air, *P* = 0.57; isocapnic hypoxia, *P* = 0.3) (see Fig. 2) were not different. Carotid body-mediated activation of ventilation (*P* = 0.73) and carotid body chemoreflex-mediated inhibition of ventilation (*P* = 0.56) were also not different between days (see Fig. 3).

**Table 4.** Cardiorespiratory parameters during room air and isocapnic hypoxia (40 mmHg)

	Room Air			Hypoxia			Hypoxia-Room Air		
	Placebo	Pentoxifylline	*P* value	Placebo	Pentoxifylline	*P* value	Placebo	Pentoxifylline	*P* value
Hemodynamics									
Heart rate, beats/min	77 ± 11	71 ± 11	0.031	86 ± 11	83 ± 11	0.92	8 ± 5	12 ± 8	0.11
Mean arterial pressure, mmHg	89 ± 6	88 ± 8	0.70	92 ± 8	93 ± 7	0.98	3 ± 4	6 ± 5	0.13
Systolic arterial pressure, mmHg	120 ± 9	119 ± 9	0.84	126 ± 9	128 ± 10	0.78	9 ± 6	10 ± 6	0.08
Diastolic arterial pressure, mmHg	74 ± 7	72 ± 7	0.75	92 ± 7	93 ± 7	0.97	19 ± 5	21 ± 5	0.45
Cardiac output, L/min	5.9 ± 1.1	5.6 ± 1.2	0.81	6.8 ± 1.3	6.7 ± 1.5	0.99	0.8 ± 1.0	1.2 ± 1.6	0.51
Stroke volume	78 ± 15	79 ± 16	0.94	79 ± 14	78 ± 15	0.98	2 ± 9	3 ± 14	0.93
Respiration									
$\text{P}E{\text{T}}_{{\text{O}}_{2}}$, Torr	52 ± 3	53 ± 2	0.67	40 ± 1	39 ± 1	0.56	−13 ± 3	−15 ± 2	0.32
$\text{P}E{\text{T}}_{\text{C}{\text{O}}_{2}}$, Torr	29 ± 3	29 ± 2	0.96	29 ± 3	29 ± 2	0.8	−0.02 ± 0.56	−0.02 ± 0.61	0.59
Peripheral oxygen saturation, %	90 ± 3	90 ± 2	0.42	76 ± 3	75 ± 3	0.81	−14 ± 3	−16 ± 3	0.43
V̇e, L/min	14.5 ± 3.8	15.6 ± 3.3	0.84	30.9 ± 16.2	32.9 ± 13.1	0.91	16.3 ± 13.8	17.2 ± 11.3	0.74
Cerebral blood flow, mL/min									
ICA flow,	247 ± 47	251 ± 34	0.96	289 ± 62	294 ± 50	0.50	42 ± 30	43 ± 42	0.92
VA flow, mL/min	89 ± 40	83 ± 36	0.49	117 ± 53	113 ± 51	0.79	27 ± 16	29 ± 14	0.59

Values are means ± SD unless otherwise specified. $\text{P}E{\text{T}}_{{\text{O}}_{2}}$, end-tidal oxygen partial pressure; $\text{P}E{\text{T}}_{\text{C}{\text{O}}_{2}}$, end-tidal carbon dioxide partial pressure; V̇e, minute ventilation; Ica, internal carotid artery and VA, vertebral artery. A paired two-tailed student’s *t* tests were used for the statistical tests.

**Figure 4.**
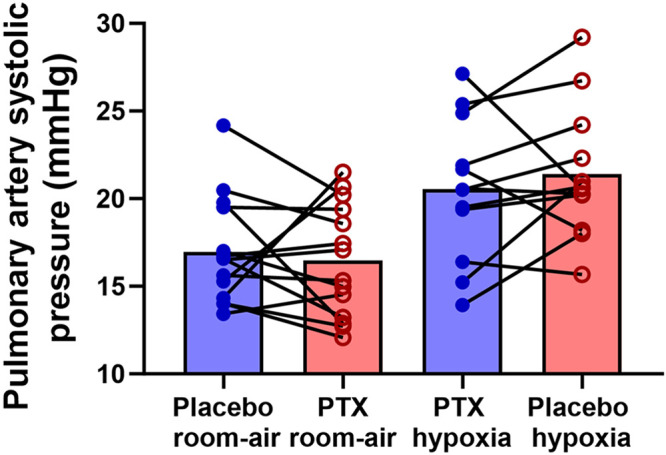
Pulmonary artery systolic pressure with placebo (blue) and pentoxifylline (PTX; red) during room air (*n* = 14) and isocapnic hypoxia (40 mmHg; *n* = 12). Closed dots are individual responses, and bar graphs are group means. Paired 2-tailed Student’s *t* tests were used for the statistical comparisons.

**Figure 5.**
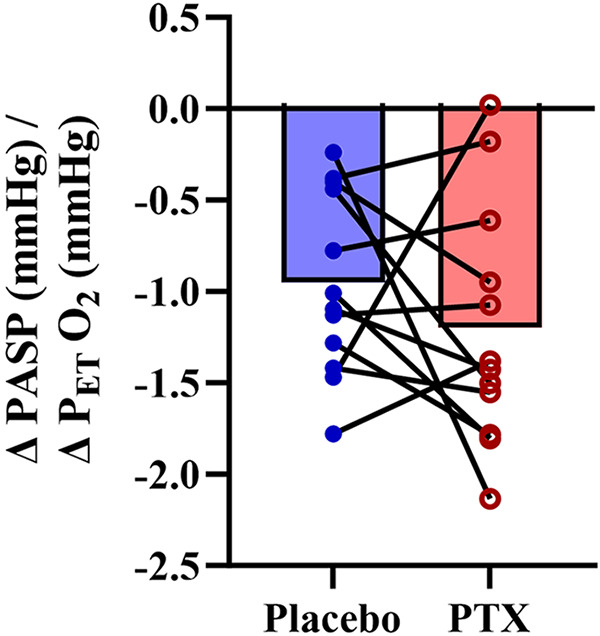
Pulmonary artery systolic pressure (PASP) sensitivity to isocapnic hypoxia (40 mmHg). The delta increase in PASP divided by the delta decrease in the end-tidal partial pressure of oxygen ($\text{P}E{\text{T}}_{{\text{O}}_{2}}$) with placebo (blue) and pentoxifylline (PTX; red). Closed dots are individual responses, and bar graphs are group means. Paired 2-tailed Student’s *t* tests were used for the statistical comparisons (*n* = 12).

**Figure 6.**
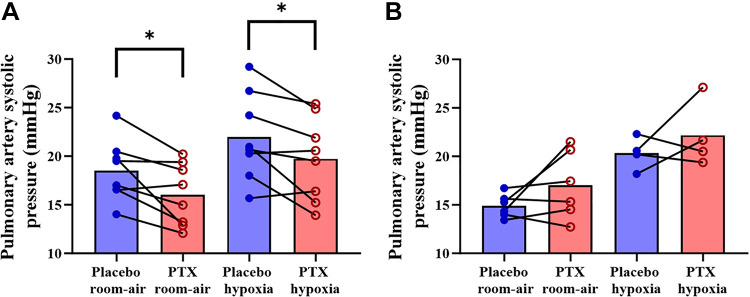
Pulmonary artery systolic pressure with placebo (blue) and pentoxifylline (PTX; red) during room air and isocapnic hypoxia (40 mmHg) in females (*A*) (room air and isocapnic hypoxia, *n* = 8) and males (*B*) (room air, *n* = 6; isocapnic hypoxia, *n* = 4). Closed dots are individual responses, and bar graphs are group means. Paired 2-tailed Student’s *t* tests were used for the statistical comparisons. *P < 0.05, placebo vs. PTX.

There was no difference between experimental days for pulmonary artery systolic pressure during room air (*P* = 0.94) or isocapnic hypoxia (*P* = 0.98) (see Fig. 4), nor was there a change in pulmonary artery systolic pressure reactivity (*P* = 0.33) (see Fig. 5). However, for the exploratory sex differences, we observed a significant interaction between sex and drug (*P* = 0.00042), but no other significant sex interactions. Females exhibited lower pulmonary artery systolic pressure during both room air (*P* = 0.021) and isocapnic hypoxia (*P* = 0.029) when given pentoxifylline compared with placebo (Fig. 6). There was also a notable effect size for room air [(pentoxifylline − placebo) effect size = 1.05] and isocapnic hypoxia [(pentoxifylline − placebo) effect size = 0.97].

## DISCUSSION

We characterized the acute impact of oral pentoxifylline on blood rheology and the pulmonary vascular, cerebrovascular, and ventilatory responses to hypoxia following 11–14 days of high-altitude (3,800 m) acclimatization. The acute administration of pentoxifylline did not alter arterial blood gases, whole blood viscosity, or red blood cell deformability, nor did it affect hypoxic cerebral or ventilatory responses. Nevertheless, it yielded an intriguing finding: a reduction in pulmonary artery systolic pressure in females, with no such effect observed in males. These results indicate that, in young, healthy participants sojourning to 3,800 m, acute administration of pentoxifylline does not influence the acclimatization process but may have sex-specific effects on pulmonary artery pressure.

Like previous findings (13), high altitude caused a significant decrease in red blood cell deformability; however, pentoxifylline failed to rectify this decline. Pentoxifylline had no effect on whole blood and plasma viscosity, red blood cell deformability, or red blood cell aggregation. Similarly, hikers sojourning to altitudes of 4,500 (18) and 4,600 m (28) while taking pentoxifylline (at a dose of 400 mg, once daily) for 5 wk reported no significant differences in blood viscosity between the control and pentoxifylline groups. However, pentoxifylline administrated at 5,150 m (400 mg 3 times daily for 3 wk) (18) and 7,350 m (400 mg once daily for 6 wk) (41) attenuated the decline in red blood cell filterability following high-altitude exposure. It is therefore plausible that pentoxifylline at high altitude does not increase red blood cell deformability per se, but negates the decline in red blood cell deformability. Red blood cell deformability declines with greater altitude (13) and hypoxia (14).

The absence of any hemorheological benefits of pentoxifylline, despite the elevations in blood viscosity and worsened deformability compared with sea level, may be due to the acute administration. Rheological benefits are often observed following months of usage. For example, blood viscosity in patients with peripheral artery disease is decreased following 1 mo of pentoxifylline administration (400 mg, 3 times daily) and further decreased following continued usage up to 2 mo (42). Pentoxifylline may have greater benefits for blood rheology at higher altitudes used for a longer period as previously demonstrated (18, 41).

Nonhuman animal studies have demonstrated that administration of pentoxifylline causes blunted hypoxic pulmonary vasoconstrictive responsiveness (19, 43–45) both in isolated lung models (44) and anesthetized dogs (43). Humans, however, have demonstrated both decreased pulmonary circulation tone (21) and reactivity (46). Notably, we observed no change in reactivity, but a change in pulmonary artery tone in females. This observed difference between males and females could be attributed to variations in plasma concentrations. Although we used a standardized oral dose (i.e., 400 mg), to ensure clinical applicability, this approach may have inadvertently resulted in females receiving a relatively higher dose than males (47) (females, 69 ± 11 kg; males, 73 ± 9 kg). Furthermore, females have smaller lungs (48) and pulmonary capillary blood volumes (49) compared with age- and height-matched males. Pentoxifylline may therefore have a greater impact on the smaller pulmonary vasculature of females. Interestingly, to our knowledge, no study has specifically investigated the effect of synthetic methylxanthine on pulmonary artery pressure in relation to sex. Pentoxifylline decreases pulmonary artery pressure via nitric oxide. In cats, *N*^ω^-nitro-l-arginine methyl ester (l-NAME) has been shown to attenuate pulmonary artery vasodilation with pentoxifylline (50). Treatment of pulmonary hypertension is more effective in males than females with phosphodiesterase inhibitors (i.e., tadalafil), which is nitric oxide dependent (51), making it improbable that differences in sex hormones are the sole cause of this sex-specific response. However, sex-related hormones cannot be entirely ruled out as a contributing factor. Hence, the possibility arises that pentoxifylline might impact the pulmonary vasculature differently in females compared with males independent of body and pulmonary size. It is also important to recognize the rather small number of males within this cohort and that the aim of this study was not to investigate sex differences. Further studies are needed to confirm this sex-specific interaction; however, these findings are promising.

Pentoxifylline has been shown to not only increase ventilation but also enhance oxygen diffusion in chronic obstructive pulmonary disorder (52) and increase p50 in anesthetized dogs (43). These effects may provide additional potential avenues for improved arterial saturation at high altitude beyond ventilation. However, our findings within this cohort do not align with this notion. It is conceivable that a longer administration of pentoxifylline could have resulted in a more pronounced impact on these mechanisms (28). However, the exact mechanisms causing this augmented arterial saturation remain unknown, warranting further investigation. We hypothesized that minute ventilation might reduce cerebral blood flow by reducing arterial carbon dioxide levels and increasing arterial content (29). However, likely because there were no significant alterations in arterial blood gases or arterial content, we observed no discernible change in cerebral blood flow when pentoxifylline was administered. A previous study demonstrated that pentoxifylline attenuates the decline in cerebral blood flow during acute hypobaric hypoxia (decompression chamber, 4,000 m) in healthy participants (27). However, similar to our findings, Wood and Appenzeller (18) found that pentoxifylline compared with control did not change middle cerebral artery velocity in mountaineers at 5,150 m (53). Considering that pentoxifylline enhances cerebral blood flow by improving red blood cell deformability, it is reasonable to propose that the absence of changes in red blood cell deformability led to the lack of subsequent alterations in cerebral blood flow. Similarly, the absence of pentoxifylline on inflammation (as indexed via TNF-α) may also explain why cerebral blood flow was unchanged (54). Importantly, we only assessed the inflammatory cytokine, TNF-α, because pentoxifylline has been shown to reduce inflammation via sustained cyclic adenosine monophosphate activation of protein kinase A and, in turn, the primary transcription factor for TNF-α, nuclear factor-κB (55). Without an observed change in TNF-α, the primary cytokine implicated in pentoxifylline’s anti-inflammatory potential, we did not feel the need to expand the inflammation panel for the present study. However, it should be noted that other inflammatory pathways, particularly concerning certain interleukins (IL; e.g., IL-6, IL-8, and IL-1), have been shown to be involved in the pathogenesis of high-altitude illness and, despite the good health of our participants, may be a precursor of such high-altitude illness and should be explored in the context of pentoxifylline administration for future studies (56–58).

Our cohort of participants were acclimatized lowlanders who were in good health (e.g., low systemic concentrations of TNF-α) without high-altitude illness. It is therefore plausible that pentoxifylline could confer greater advantages to individuals burdened with altitude-related illnesses characterized by hyperviscosity, high levels of inflammation, exaggerated hypoxemia, and pulmonary vascular pressures (i.e., chronic mountain sickness and high-altitude pulmonary edema). It therefore seems logical to consider using pentoxifylline at higher altitudes for a longer duration of time. Such an approach could potentially yield more substantial benefits, especially given that pentoxifylline is inexpensive and widely available.

### Conclusions

Acute administration of pentoxifylline in healthy, acclimatized lowlanders did not alter arterial blood gases, whole blood viscosity, red blood cell deformability or aggregation, inflammation, cerebral blood flow, or ventilation at 3,800 m. However, an unexpected and potentially unique finding emerged, as the treatment resulted in a reduction in pulmonary artery pressure in female participants, while no such effect was observed in males. Further investigation is warranted to confirm and elucidate the mechanism that might determine this sex-specific response.

## DATA AVAILABILITY

Data are available upon request from the corresponding author.

## GRANTS

Funding for this study was received by Gouvernement du Canad-Natural Sciences and Engineering Research Council of Canada and Principal’s Research Chair (to P.N.A.).

## DISCLOSURES

No conflicts of interest, financial or otherwise, are declared by the authors.

## AUTHOR CONTRIBUTIONS

A.R.S., C.A.H., T.D.G., J.D.A., P.N.A., and J.C.T. conceived and designed research; C.A.H., T.D.G., K.F., A.M.W., H.G.C., L.M.B., J.D., J.A.M., P.S., J.D.A., and J.C.T. performed experiments; A.R.S., C.A.H., A.M.W., and J.C.T. analyzed data; A.R.S., C.A.H., T.D.G., K.F., A.M.W., H.G.C., L.M.B., J.D., J.A.M., P.S., J.D.A., M.S., P.N.A., and J.C.T. interpreted results of experiments; A.R.S. and J.C.T. prepared figures; A.R.S. and J.C.T. drafted manuscript; A.R.S., C.A.H., T.D.G., K.F., A.M.W., H.G.C., L.M.B., J.D., J.A.M., P.S., J.D.A., M.S., P.N.A., and J.C.T. edited and revised manuscript; A.R.S., C.A.H., T.D.G., K.F., A.M.W., H.G.C., L.M.B., J.D., J.A.M., P.S., J.D.A., M.S., P.N.A., and J.C.T. approved final version of manuscript.
